# Role of Mast Cells in Inflammatory Bowel Disease and Inflammation-Associated Colorectal Neoplasia in IL-10-Deficient Mice

**DOI:** 10.1371/journal.pone.0012220

**Published:** 2010-08-17

**Authors:** Maciej Chichlowski, Greg S. Westwood, Soman N. Abraham, Laura P. Hale

**Affiliations:** 1 Department of Pathology, Duke University Medical Center, Durham, North Carolina, United States of America; 2 Department of Molecular Genetics and Microbiology, Duke University Medical Center, Durham, North Carolina, United States of America; 3 Department of Immunology, Duke University Medical Center, Durham, North Carolina, United States of America; 4 The Human Vaccine Institute, Duke University Medical Center, Durham, North Carolina, United States of America; New York University, United States of America

## Abstract

**Background:**

Inflammatory bowel disease (IBD) is hypothesized to result from stimulation of immune responses against resident intestinal bacteria within a genetically susceptible host. Mast cells may play a critical role in IBD pathogenesis, since they are typically located just beneath the intestinal mucosal barrier and can be activated by bacterial antigens.

**Methodology/Principal Findings:**

This study investigated effects of mast cells on inflammation and associated neoplasia in IBD-susceptible interleukin (IL)-10-deficient mice with and without mast cells. IL-10-deficient mast cells produced more pro-inflammatory cytokines *in vitro* both constitutively and when triggered, compared with wild type mast cells. However despite this enhanced *in vitro* response, mast cell-sufficient *Il10* ^−/−^ mice actually had decreased cecal expression of tumor necrosis factor (TNF) and interferon (IFN)-γ mRNA, suggesting that mast cells regulate inflammation *in vivo*. Mast cell deficiency predisposed *Il10* ^−*/*−^ mice to the development of spontaneous colitis and resulted in increased intestinal permeability *in vivo* that preceded the development of colon inflammation. However, mast cell deficiency did not affect the severity of IBD triggered by non-steroidal anti-inflammatory agents (NSAID) exposure or helicobacter infection that also affect intestinal permeability.

**Conclusions/Significance:**

Mast cells thus appear to have a primarily protective role within the colonic microenvironment by enhancing the efficacy of the mucosal barrier. In addition, although mast cells were previously implicated in progression of sporadic colon cancers, mast cells did not affect the incidence or severity of colonic neoplasia in this inflammation-associated model.

## Introduction

Inflammatory bowel disease (IBD) is characterized by aberrant immune responses against microorganisms that are present in the intestine. Debilitating clinical symptoms of pain and diarrhea result from intestinal mucosal damage that is driven by the continuous activation of the mucosal immune system by enteric bacteria. A variety of genetic, microbial, and environmental factors have been identified that increase susceptibility to IBD in both animal models and humans. Based on this data, we have proposed that the development of IBD requires three factors [1]. First, bacterial antigens and adjuvants must be present within the intestine. This factor is not easily modifiable, since potentially colitogenic bacterial antigens and adjuvants are present within the intestine of all humans and all mice that are not kept in germ-free facilities. Second, the mucosal barrier must be defective so that the bacterial antigens and adjuvants present within the intestine can come in contact with the innate and adaptive immune cells to generate responses. And third, the host must have a defect in immune regulation that allows induction of sustained immune responses against these antigens. This three-factor model can potentially explain how the known susceptibility alleles and IBD-related triggers in existing murine models result in the development of chronic colitis. The model also predicts that colitis can potentially be prevented or treated by interventions that favor maintenance of appropriate immune regulation and/or enhancement of mucosal barrier function.

Most of the currently used IBD treatments target the immune regulatory pathways, but resulting immunosuppression can increase patient risk for developing opportunistic infections and/or treatment-related lymphomas. Mechanisms that govern the barrier function of the intestinal mucosa are thus of great interest to identify potential targets for novel IBD therapies that can add or synergize with existing therapies. A number of murine models of intestinal inflammation have been established [1], [2]. One very commonly used model uses mice deficient in interleukin (IL)-10. These mice have defects in immune regulation and develop chronic enterocolitis with loss of tolerance to bacterial stimuli when triggered by environmental exposures that decrease mucosal barrier function [3], [4], [5], [6].

Mast cells are innate immune cells that can potentially contribute to IBD through their pro- inflammatory activity and/or effects on immunoregulation. Their pattern recognition molecules allow them to readily recognize and rapidly respond to bacteria that breach the epithelium [7]. Upon activation, mast cells can immediately release large amounts of pro-inflammatory cytokines that are contained in pre-formed granules [8] and can continue to synthesize and release a wide array of pro-inflammatory mediators *de novo*. Mast cells thus rapidly and selectively produce appropriate mediators that enhance effector-cell recruitment and complement other effector components of the immune system [9]. For example, mast cell-derived mediators can contribute to colitis severity by enhancing neutrophil influx and thus perpetuating ongoing inflammation. However mast cells have also been documented to have anti-inflammatory or immunosuppressive functions, such that they can serve to either enhance or to limit innate or adaptive immune responses, depending on the context [10].

Mast cells are physically located adjacent to the intestinal epithelium, so their activation may also affect the function of the mucosal barrier. The presence of mast cells or the mast cell-produced proteinase *Mcpt4* (chymase) was recently shown to enhance the permeability of jejunal segments studied *ex vivo* [8]. The same study showed that mast cell-deficient Kit^W-sh/W-sh^ (sash) mice have changes in small intestinal architecture, including increased crypt depth, decreased migration of epithelial cells up the villus, and decreased expression of the tight junction protein claudin-3 compared to wild type mice [8]. Claudin-3 is an important sealing protein and its loss from colon tissue has been correlated with lack of tight junction integrity [11]. Mast cells have also been shown to mediate increased intestinal permeability caused by exposure to stress neuropeptides (e.g. corticotropin-releasing factor or sauvagine) *in vitro* [12]. Several studies have shown increased numbers of mast cells or increased release of mast cell mediators from actively inflamed colon of IBD patients compared with non-inflamed colon or normal controls [13], [14], [15], [16], suggesting a potential role for mast cells in the pathogenesis of IBD. However, specific *in vivo* data relating to mechanisms by which mast cells may influence IBD pathogenesis remains limited.

In view of the the well known role of mast cells in exacerbating inflammatory diseases such as arthritis, allergy, and asthma [17], [18], [19] and the potential of mast cells to contribute to one or more of the three factors that affect development of IBD, we hypothesized that mast cells might play a prominent role in the pathogenesis of IBD. We used a well-established model of IBD, based on IL-10-deficient mice with and without added mast cell deficiency to test this hypothesis.

## Results

### Mediator response is elevated in Il10^−/−^ mast cells, but absence of mast cells does not affect the severity of IBD triggered by piroxicam in Il10^−/−^ mice

**Figure 1:**
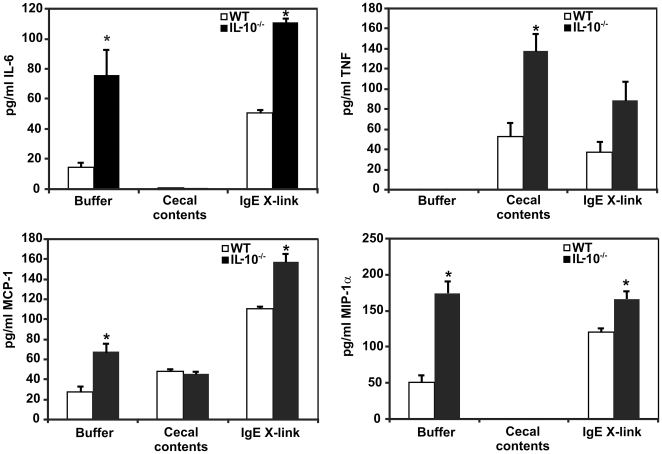
BMMC cytokine production after stimulation with IgE or enteric bacteria. BMMC were stimulated for 4 hrs, then media was harvested for cytokine analysis via Luminex bead-based fluorescent immunoassays. *Il10* ^−*/*−^ BMMC make more IL-6, MCP-1, and MIP-1α constitutively (buffer treatment) and following IgE stimulation than WT BMMC (*p*<0.05). Exposure to enteric bacteria in cecal contents triggers a different pattern of cytokine expression, with higher TNF production by *Il10* ^−/−^ vs. WT BMMC. Data shown is the mean ± SEM for 3–5 independent experiments. * indicates *p*≤0.05 vs. WT.

The purpose of these studies was to determine the role of mast cells in the pathogenesis of IBD. *Il10* ^−/−^ mice were used, since they are highly susceptible to developing IBD when subjected to conditions that enhance mucosal permeability. Since IL-10 can also directly affect the function of immune cells including mast cells [20], bone marrow-derived mast cells (BMMC) from wild-type (WT) and *Il10* ^−*/*−^ mice were first compared to determine how IL-10 deficiency affected the ability of BMMC to produce other inflammatory mediators. Similar mast cell survival and % degranulation *in vitro* were observed for WT vs. *Il10* ^−*/*−^ BMMC after the treatment with IgE + cross-linking with anti-IgE. Exposure to enteric bacteria (cecal contents) did not cause significant degranulation of either wild type or *Il10* ^−*/*−^ mast cells and also did not affect mast cell survival (data not shown). However, *Il10* ^−*/*−^ BMMC produced higher baseline levels of IL-6, MCP-1, and MIP-1α in the absence of stimulation (buffer-treated cells) than did WT BMMC (Figure 1). Levels of IL-6 and MCP-1 secretion following IgE-induced degranulation were further increased in *Il10* ^−*/*−^ BMMC compared with WT BMMC (Figure 1). Exposure of BMMC to enteric bacteria (cecal contents) triggered a pattern of cytokine production distinct from that triggered by IgE stimulation, with increased production of tumor necrosis factor (TNF) by *Il10* ^−*/*−^ compared with WT BMMC (*p*<0.05), but decreased or unchanged production of MCP-1, IL-6, and MIP-1α (Figure 1). The increased baseline and stimulated production of pro-inflammatory cytokines by *Il10* ^−/−^ BMMC to both classic (e.g. IgE) and IBD-relevant (e.g. enteric bacteria) activation stimuli *in vitro* suggested that *Il10* ^−/−^ mast cells might be particularly potent in stimulating inflammatory reactions in the gut following breakdown of the mucosal barrier *in vivo*.

**Figure 2:**
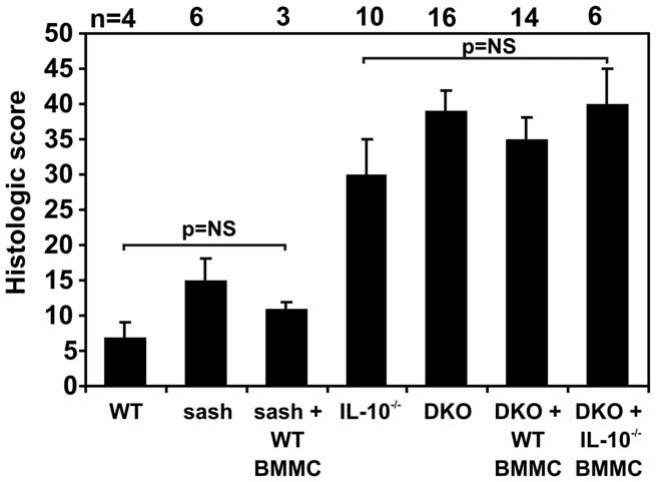
Mast cells do not affect the severity of colitis triggered by piroxicam. Mice of the indicated genotypes ± reconstitution with WT or *Il10* ^−/−^ BMMC received 200 ppm piroxicam ×7 days to trigger the onset of colitis. Tissue was harvested for histologic analysis 16 d later. WT or sash mice ± reconstitution with WT BMMC did not develop colitis in this study. Mice deficient in IL-10 uniformly developed colitis, however the presence or absence of mast cells and whether or not the mast cells could produce IL-10 did not affect the severity of inflammation.

Exposure to the non-steroidal anti-inflammatory agent (NSAID) piroxicam uniformly triggers the development of IBD in *Il10* ^−/−^ mice by enhancing apoptosis of mucosal epithelial cells, resulting in barrier breakdown that massively exposes immune cells in the lamina propria to bacterial antigens [5]. Mice variously deficient in IL-10 and mast cells received 200 ppm piroxicam for 7 days and then were observed for 16 additional days prior to assessment of colon inflammation. Wild type mice did not develop chronic colitis when exposed to piroxicam (mean histologic scores ± SEM  = 7±2; Figure 2). Mast cell-deficient sash mice also did not develop colitis following piroxicam exposure, either with or without reconstitution with WT BMMC (Figure 2). As reported previously [5], IL-10-deficient mice developed moderate to severe colitis when exposed to piroxicam (mean histologic scores ± SEM  = 30±5; Figure 2). *Il10* ^−*/*−^ mice with mast cell deficiency due to the *Kit^W-sh^/Kit^W-sh^* mutation (DKO mice), DKO mice reconstituted with WT BMMC, and DKO mice reconstituted with *Il10* ^−*/*−^ BMMC also developed moderate to severe colitis when exposed to piroxicam, with severity that statistically did not differ from that seen in mast cell-sufficient *Il10* ^−*/*−^ mice (p = 0.14, 0.34, and 0.93, respectively; Figure 2). Thus, even though *Il10* ^−/−^ mast cells produce elevated levels of inflammatory mediators when stimulated *in vitro*, the absence of mast cells does not affect the severity of IBD triggered by exposure of *Il10* ^−/−^ mice to the mucosal barrier-damaging NSAID piroxicam.

### Mast cells modulate production of pro-inflammatory mediators in vivo, but absence of mast cells does not affect the severity of helicobacter-triggered IBD in Il10^−/−^ mice

**Figure 3:**
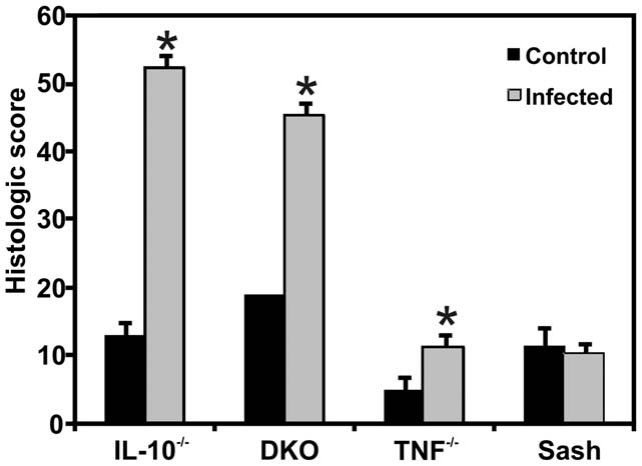
Mast cells do not affect early colitis severity in Helicobacter-infected *Il10* ^−/−^ mice. Both IL-10-deficient and DKO mice exhibited severe colonic inflammation when infected with *H. rodentium* and *H. typhlonius* for 30 d (n = 3 for *Ill0* ^−/−^ and n = 4 for DKO mice) compared to *Tnf* ^−/−^ and sash mice (n = 4 each) (*p*<0.001 for both *Il10* ^−/−^ and DKO vs. *Tnf* ^−/−^ and sash). * indicates *p*<0.01, comparing control (non-infected) and infected mice of a given genotype.

**Figure 4:**
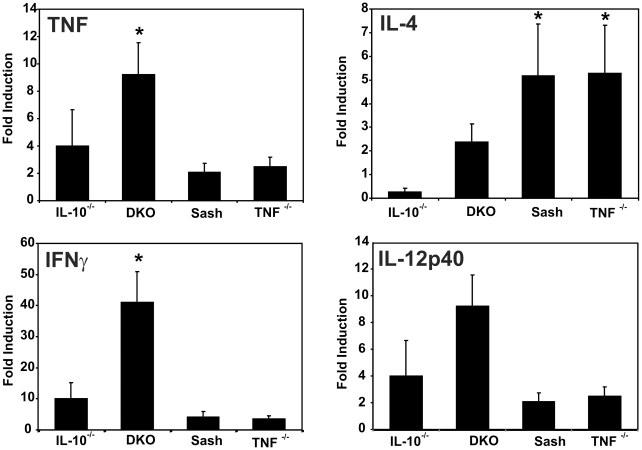
Mast cells modulate pro-inflammatory cytokine mRNA expression in inflamed cecal tissues. Cytokine mRNA expression in cecal tissue of mice of the indicated genotypes harvested after 5 weeks of infection with *H. rodentium* and *H. typhlonius* was measured by real-time PCR, normalized to *β*-actin and expressed as fold-induction relative to non-infected cecal tissue of the same genotype. Bars represent 5–6 mice per genotype. * indicates *p*<0.05 compared to *Il10* ^−/−^ mice. Infected DKO mice have increased expression of Th1 cytokines (TNF, IFN-γ) and a trend towards increased Th2 (IL-4) and Th17-inducing cytokines (IL-12/23p40) compared with mast cell-sufficient *Il10* ^−/−^ mice.

*In vivo* activation responses of mast cells may differ from their responses *in vitro* due to the complexities of the *in vivo* colonic microenvironment. To address this issue, we determined the effect of mast cells on the inflammatory milieu in IBD-susceptible and control mice with mucosal barrier compromise due to helicobacter infection. Colon tissues from *Il10* ^−*/*−^, DKO, sash, and TNF-deficient mice with and without co-infection by *H. typhlonius* and *H. rodentium* were analyzed for inflammation severity and for the production of selected cytokines by real time reverse transcriptase polymerase chain reaction (PCR) at 4–5 wks after infection. At this time point, *Il10* ^−*/*−^ and DKO mice have severe chronic colitis, while *Tnf* ^−/−^ and mast cell-deficient sash mice do not (Figure 3). DKO mice had significantly increased production of mRNA encoding TNF and interferon (IFN)-γ, with strong trends toward increased IL-4 and IL-12/23p40 compared with mast cell-sufficient *Il10* ^−*/*−^ mice (Figure 4). Elevated levels of IL-4 were also seen in sash mice singly deficient in mast cells and in TNF-deficient mice that do not develop colitis under these conditions, compared with *Il10* ^−*/*−^ mice that develop severe colitis (Figure 4). Thus, tissue from DKO mice with colitis showed increased production of pro-inflammatory cytokines compared with mice singly deficient in IL-10, mast cells, or TNF. This demonstrates that mast cells play a role in down-regulating production of pro-inflammatory cytokines within the inflamed colon, at this relatively early time point in the course of chronic colitis.

**Figure 5:**
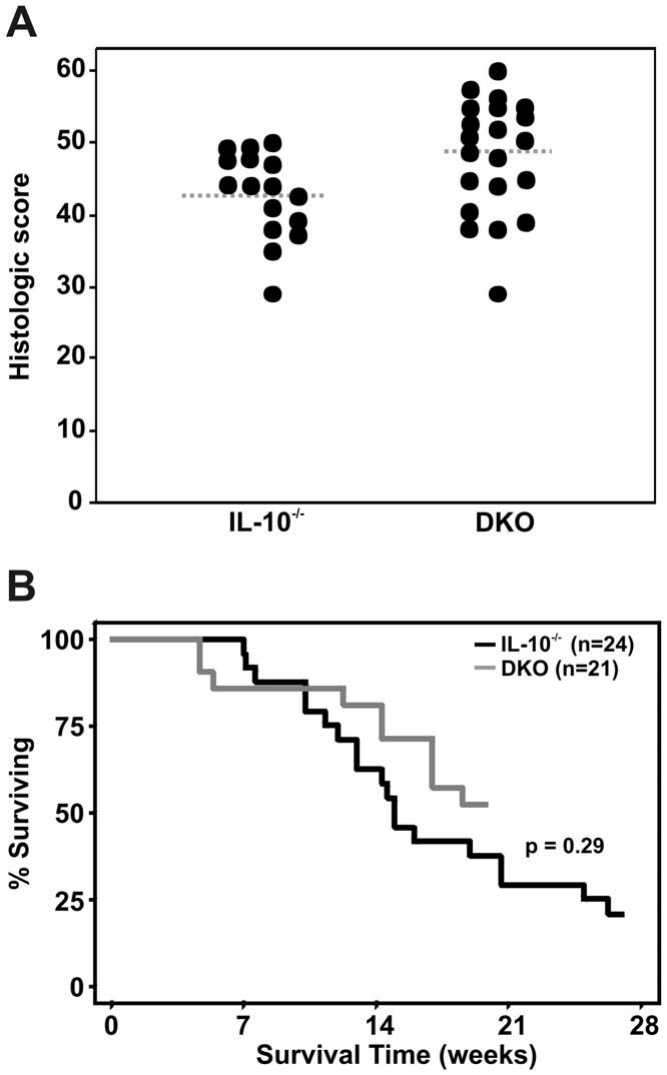
Mast cells do not affect long-term colitis severity in Helicobacter-infected *Il10* ^−/−^ mice. *Il10* ^−/−^ mice with (n = 21) and without (n = 24) genetic deficiency of mast cells were infected with *H. rodentium* and *H. typhlonius* and monitored for up to 27 wks. DKO mice had a small increase in histologic score compared with mice deficient in IL-10 alone (A; p = 0.03), but there was no difference in survival between DKO and mast cell-sufficient *Il10* ^−/−^ mice (B).

To determine how mast cells affected the long-term severity of IBD in helicobacter-infected mice that had sustained mucosal barrier dysfunction, *Il10* ^−*/*−^ and DKO mice were infected with *H. rodentium* and *H. typhlonius* and monitored for up to 27 wks post-infection. Infected *Il10* ^−/−^ and DKO mice both demonstrated severe colitis that was slightly increased in the absence of mast cells (mean histologic score  = 43±1 in *Il10* ^−/−^ mice vs. 48±2 in DKO mice; *p* = 0.03) (Figure 5A). However, this slight difference in colitis severity did not translate into a difference in survival based on presence or absence of mast cells (p = 0.29; Figure 5B).

### Mast cells do not affect inflammation-associated colonic neoplasia in helicobacter-infected Il10^−/−^ mice

**Figure 6:**
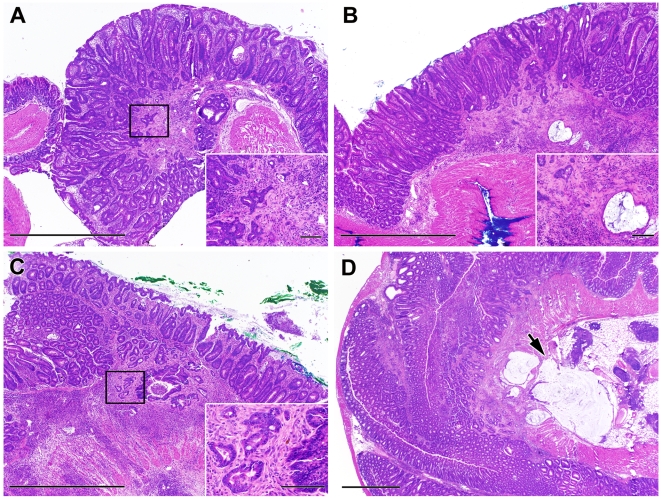
Colon histology and neoplasia in *Helicobacter*-infected and non-infected *Il10* ^−/−^ and DKO adult mice. Helicobacter-infected *Il10* ^−/−^ (A, B) and DKO mice (C, D) uniformly exhibited mucosal hyperplasia with prominent inflammatory infiltrates. Examples of neoplastic lesions seen in the long-term study are shown. The arrow in D indicates malignant glands that have invaded into the serosa. Bar represents 1 mm in the large panels and 100 µm in the insets.

Colonic inflammation has previously been shown to predispose to the development of malignancy, both in humans and in murine models of IBD [6], [21]. Mast cells have also been shown to affect the incidence and severity of neoplasia in models of sporadic colon cancers [22], [23]. Therefore, the effect of mast cells on inflammation-associated neoplasia was also investigated in *Il10* ^−/−^ mice with colitis triggered by helicobacter infection. Sixty three percent of *Il10* ^−/−^ mice (n = 24) developed colorectal neoplasia by 18±1 weeks after IBD triggering, with an average of 3±2 neoplastic lesions per mouse (range = 1–6) (Figure 6A and B). Similarly, 62% (n = 21) of DKO mice had colorectal neoplasia 17±1 weeks after IBD triggering, with an average of 2±1 neoplastic lesions per mouse (range = 1–6) (Figure 6C and D). Half of these neoplastic lesions were invasive at the time of detection in both groups. Thus mast cells do not affect the incidence of inflammation-associated neoplasia in *Il10* ^−/−^ mice with severe long-standing compromise of the mucosal barrier due to helicobacter infection.

### Absence of mast cells predisposes to spontaneous development of IBD in Il10^−/−^ mice

**Figure 7:**
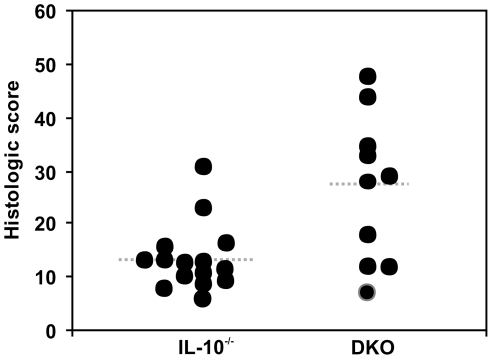
Severity of spontaneous IBD in specific pathogen-free *Il10* ^−/−^ and DKO mice. *Il10* ^−/−^ mice on the C57BL/6 background rarely developed spontaneous colitis by 36 weeks of age when housed under clean conditions, free from specific pathogens including Helicobacter *spp.* (mean histologic scores ± SEM = 13±2; n = 15). In contrast, DKO mice that lacked mast cells frequently developed spontaneous colitis (mean histologic scores = 27±4; n = 10; *p* = 0.02), which was evident at an earlier age (31±2 wks vs. 36±1 wks; p = 0.02) compared with *Il10* ^−/−^ mice.

Since we did not see any contributory role for mast cells in actively induced inflammatory responses in gut of *Il10* ^−/−^ mice, we investigated whether mast cells had a role in the spontaneous development of IBD in *Il10* ^−*/*−^ vs. DKO mice. All mice were documented to remain free of helicobacter infection throughout the study. Whereas most mast cell-sufficient *Il10* ^−*/*−^ mice did not develop colitis by 9 months of age (mean histologic score ± SEM  = 13±2), most DKO mice developed at least moderate colitis (mean ± SEM histologic score  = 27±4) (Figure 7). These DKO mice were significantly younger (31±2 wks; range  = 25–38 wks; n = 10) than the *Il10* ^−*/*−^ mice studied (36±1 wks; range 34–36 wks; n = 15; p = 0.02) due to more frequent occurrence of rectal prolapse (an indicator of severe IBD) in DKO mice that led to euthanasia for humane reasons prior to the planned study endpoint. Overall, these results demonstrate that mast cells protect *Il10* ^−/−^ mice against the development of spontaneous colitis.

### Mast cells enhance epithelial barrier function

*Il10* ^−/−^ mice have defects in immune regulation due to their genetic deficiency of IL-10 and their intestines contain potentially colitogenic antigens and adjuvants derived from their commensal intestinal microbiota. The three factor model presented earlier thus predicts that colitis will develop when the mucosal barrier is compromised, a prediction supported by the ability of NSAIDs and helicobacter infection to trigger colitis in these mice. Our finding that absence of mast cells predisposed to spontaneous development of IBD in *Il10* ^−/−^ mice suggested that absence of mast cells might also decrease the effectiveness of the mucosal barrier.

**Figure 8:**
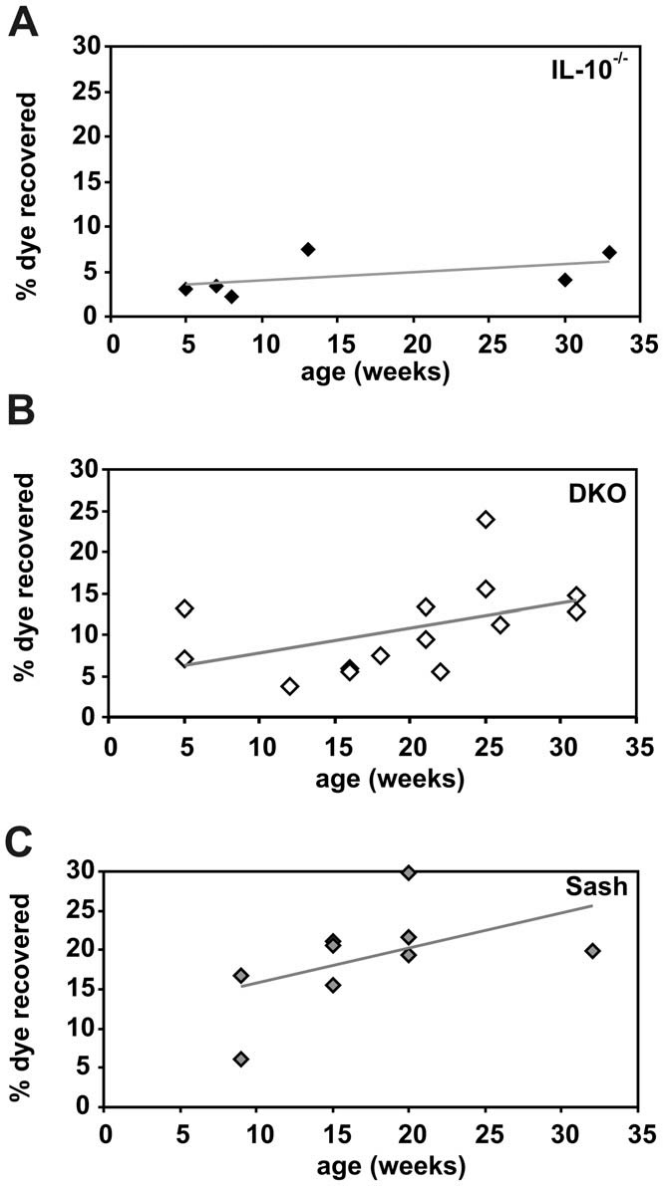
Permeability of the intestinal barrier in IL-10^−/−^ and DKO mice. Mice of the indicated ages and genotypes were gavaged with phenol red and the amount of dye absorbed was determined by spectrophotometric measurement of urine. Each point shown represents the average of 2–4 mice tested in a single experiment. Mast cell-deficient DKO and sash mice had significantly increased dye recovery compared with *Il10* ^−/−^ mice (p = 0.02 for differences in data point elevation via linear regression analysis). The total numbers of mice tested were: *Il10* ^−/−^ (n = 14), DKO (n = 18), and sash (n = 22).

A quantitative assessment of barrier function was performed to determine the effect of mast cells on intestinal permeability. Phenol red dye was administered orally to *Il10* ^−/−^ vs. DKO mice *in vivo* and the amount of dye recovered in urine was measured as an indicator of intestinal paracellular permeability. DKO mice exhibited higher permeability as indicated by increased dye recovery (Figure 8B) compared to *Il10* ^−*/*−^ mice (Figure 8A; p = 0.02). Histologic scores indicated minimal to no inflammation in the animals tested (mean histologic score ± SEM = 7±1 (n = 33) for DKO and 15±3 (n = 18) for *Il10* ^−*/*−^ mice). The increased intestinal permeability of DKO mice was due to the *Kit^W-sh^* mutation that results in absence of mast cells, since it was also seen in sash mice that lacked mast cells, but were wild type with respect to IL-10 expression (Figure 8C). Taken together, these studies indicate that mice that lack mast cells have increased intestinal permeability. Furthermore, the data clearly show that the decreased barrier integrity observed in DKO relative to *Il10* ^−/−^ mice occurs prior to, rather than in response to, the development of colon inflammation.

## Discussion

Although mast cells are best known for their pathogenic over-responses in immune-mediated diseases such as allergy and asthma, their protective role in innate immune responses is underscored by high susceptibility of mice deficient in mast cells to fatal infections with enteric bacteria [24], [25]. Mast cells aid the development of antigen-specific cellular and humoral adaptive immune responses by stimulating lymph node swelling and sequestration [26]. The data presented here suggest that mast cells also enhance intestinal mucosal barrier function *in vivo* and can protect against spontaneous development of IBD in susceptible mouse strains.

Intestinal epithelial barrier function is regulated by epithelial cells, innate and adaptive immunity, and the enteric nervous system and dysregulation of any of these alters the barrier [27]. Several mast cell-derived mediators, including histamine, serotonin, arachidonic acid, and mast cell proteinases have been shown to affect intestinal epithelial function, including ion channel conductance and activation/inhibition of secretion of electrolytes and mucus [8], [27], [28]. Our results show that mast cell-deficient sash and DKO mice had increased the baseline paracellular permeability of the intestine *in vivo*, independent of IL-10 status (Figure 8).

In addition to increased intestinal permeability, the data presented also show that mast cell-deficient DKO mice are at increased risk for spontaneous development of IBD compared to mast cell-sufficient *Il10* ^−/−^ mice. These results parallel previous studies in humans demonstrating that patients with IBD and 10–25% of their first degree relatives have increased paracellular permeability relative to controls and that relapse of quiescent IBD was often preceded by increased intestinal permeability [29], [30], [31]. Interestingly, the *Il10* ^−/−^ mice in our study had low gut permeability even at the relatively advanced ages of 30–35 wks. Others have shown that *Il10* ^−/−^ mice develop increased gut permeability prior to development of colitis [32]. However, the lack of barrier dysfunction in the 30–35 wk old *Il10* ^−/−^ mice reported here (Figure 8) correlates very well with the low incidence of spontaneous colitis (2 of 15 = 13%) that we observed in 36 wk old *Il10* ^−/−^ mice (Figure 7).

A recent report by Groschwitz *et al* [8] showed that jejunal tissue from mast cell-deficient mice had *decreased* baseline permeability *ex vivo* compared to jejunal tissue from wild type mice. Our data differs since it represents an integration of the permeability of non-manipulated tissues throughout the gastrointestinal tract of live mice. The permeability characteristics of small intestine and colon are known to be different with respect to water as well as a variety of nutrients. Our *in vivo* studies also include potential contributions from different mast cell subsets that may be present in different parts of the intestinal tract, bacterial-mucosal interactions, leukocyte trafficking, and transient inflammation that can also affect net intestinal permeability *in vivo*. It is also possible that mast cells contribute differentially to homeostatic versus inflammation-induced barrier function. For example, during the normal non-inflamed state, mast cells may increase permeability (as reported by Groschwitz *et al*. [8]) to optimize small intestinal absorption of nutrients and antigens and to facilitate cellular interaction important for both digestive and immune system functions. However, mast cells may limit inflammation-induced barrier dysfunction by rapidly responding to pathogens that cross the mucosal barrier. The specific types of mast cells present and the quantity and type of mediators they release upon activation are likely to be critical in regulating their net effect [33].

We found that mast cell-sufficient *Il10* ^−/−^ mice demonstrated decreased mucosal production of several pro-inflammatory cytokines (TNF, IFNγ, and IL12/23p40) compared with mice singly deficient in IL-10, mast cells, or TNF (Figure 4). Several previous studies using murine models have shown these pro-inflammatory cytokines can directly increase intestinal epithelial permeability by inducing disassembly of epithelial cell tight junctions (reviewed in [1]). Based on current understanding of tight junction function, the decrease in claudin-3 documented in intestinal tissues from mast cell-deficient sash mice [8] would be expected to increase the *in vivo* permeability of their intestinal tissues *in vivo* [11]. Perturbation of tight junctions and increased mucosal permeability and activation of the mucosal immune system have been shown to affect the severity of mucosal inflammation in humans [34], [35], [36]. However, since effect of mast cells on the mucosal barrier likely results from a balance of multiple effector and immunoregulatory functions [10], more studies will be required to precisely determine the mechanisms involved.

The Kit^W-sh^ allele that renders sash mice mast cell-deficient contains an inversion that abolishes Kit expression in mast cells and melanocytes but does not affect Kit expression in most other cell types [37]. Recent work has further identified that this inversion also inhibits the production of corin, a proteinase responsible for the activation of atrial natriuretic peptide [38]. It is also possible that the increased permeability may be due to this or to other factors in sash mice that render them susceptible to inflammation independent of their mast cell deficiency. However, we note that, despite their increased intestinal permeability, mice bearing the Kit^W-sh^ mutation alone do not have added susceptibility to IBD in the absence of concomitant IL-10 deficiency. Furthermore, reconstitution of *Il10* ^−/−^ mice also homozygous for the Kit^W-sh^ mutation (DKO) mice with mast cells does not affect IBD severity (Figures 2 and 3).

Since mast cells protect *Il10* ^−/−^ mice from spontaneous IBD, their failure to influence the severity of inflammation triggered by exposure of *Il10* ^−*/*−^ mice to piroxicam or co-infection with *H. rodentium* and *H. typhlonius* may seem paradoxical at first glance. However, this result is predicted by the three factor model of IBD pathogenesis presented earlier [1]. *Il10* ^−/−^ mice have bacteria within their intestine and their IL-10 deficiency results in defective immunoregulation. The model thus predicts that colitis will develop when the mucosal barrier is compromised. Exposure to piroxicam or infection with helicobacter already compromises mucosal barrier sufficiently to result in colitis. Thus further compromise of the barrier by mast cell deficiency would be predicted to have little to no additional effect, as was observed experimentally. Additional studies using mice with mast cell deficiency and/or alterations in intestinal permeability derived via other mechanisms will be useful for further confirming (or disproving) this hypothesized mechanism.

We also found that the presence or absence of mast cells had no effect on the incidence or progression of inflammation-associated colon cancers in *Il10* ^−/−^ mice with colitis. There is currently controversy in the literature regarding the role of mast cells in tumor progression in the colon. Mice with a genetic susceptibility to intestinal neoplasia (Apc*^Min^*) had a greater frequency and size of adenomas in the absence of mast cells, suggesting a protective function for mast cells in colorectal carcinogenesis [23]. However, another report [22] showed recently that mast cells were required for the growth of adenomatous polyps in mice. Of note, both of these studies used different strains of mice genetically susceptible to polyp formation in models with minimal inflammation. Mast cells may thus have varying effects on the inflammatory milieu in these models based on any additional environmental triggers of intestinal inflammation that may be present.

In summary, our studies suggest that mast cells have primarily a protective role within the colonic microenvironment, serving to enhance barrier function and limit spontaneous inflammation. However, the presence of mast cells is insufficient to affect the severity of IBD triggered by massive disruption of the mucosal barrier and does not affect the development and progression of colorectal neoplasia associated with severe IBD.

## Materials and Methods

### Ethics Statement

All animal studies were approved by the Institutional Animal Care and Use Committee of Duke University, an institution accredited by the Association for Assessment and Accreditation of Laboratory Animal Care (AAALAC) International. The protocol numbers covering this work were A225-06-07 and A151-09-05.

### Animal Studies

Breeding pairs of IL-10-deficient mice (strain name = *B6.129P2-Il10^tm1Cgn^/J*; stock # 002251), sash mice (strain name = *B6.Cg-Kit^W-sh^/HNihrJaeBsmJ*; stock #005051), mice transgenic for global expression of green fluorescent protein (GFP) (strain name = *C57BL/6-Tg(UBC-GFP)30Scha/J*; stock # 004353), and mice deficient in TNF (strain name = *B6.129S6-Tnf^tm1Gkl^/J*; stock # 005540) were obtained from Jackson Laboratories (Bar Harbor, ME). IL-10-deficient mice have been documented to be highly susceptible to developing IBD, either spontaneously or in response to triggers such as infection with intestinal helicobacter species or exposure to NSAIDs that damage their mucosal barrier [4], [5], [6], [39]. The Kit^W-sh^ allele contains an inversion located proximal to the Kit locus that abolishes Kit expression in mast cells and melanocytes but does not affect Kit expression in germ cells or erythrocytes [37]. Thus, although sash mice are white and lack mast cells, they can be propagated as homozygotes with normal litter sizes [40]. *Il10* ^−/−^ and sash strains were crossed to generate DKO mice that were IBD-susceptible and lacked mast cells. All of these strains were on the C57BL/6 background.

Mice were housed in polycarbonate micro-isolator cages in individually ventilated racks under BSL-2 conditions, with access to food and water *ad libitum*. Mice were observed daily for clinical signs of distress and weight was monitored three times per week. Humane endpoints included >15% loss of body weight and development of rectal prolapse, a well-recognized complication of chronic inflammation in the colon.

Sentinel mice exposed repetitively to dirty bedding from the mice used in this study were negative for parasites by microscopic exam, negative for *Citrobacter rodentium* by fecal culture, and negative by serology for a panel of 22 murine protozoal, bacterial, and viral pathogens, including murine parvovirus, murine hepatitis virus, and murine norovirus. All mice not intentionally infected with Helicobacter *spp.* were confirmed to be Helicobacter-free by PCR of feces using a species-specific primer.

### Models of Inflammatory Bowel Disease

For studies of spontaneous colitis, cohorts of mice were housed under specific pathogen-free conditions and euthanized for histologic scoring of colon inflammation if they experienced >15% loss of body weight, rectal prolapse, or reached pre-determined time points. Some cohorts of mice were exposed to 200 ppm piroxicam in powdered LabDiet 5001 chow (Purina, Framingham, MA) for 7 days to trigger the onset of colitis as previously described [6]. Based on measured food consumption, the dose of piroxicam averaged 40 mg/kg/day (range 21–62 mg/kg/day over 35 cage-days). The mice were then placed back on pelleted 5001 chow without piroxicam and observed for an additional 16 days before euthanasia for histologic scoring of colon inflammation. For studies of colitis triggered by helicobacter infection, 6–8 wk old mice were infected with *H. typhlonius* (clinical isolate DU-01) [41] and *H. rodentium* (MIT 95–1707 =  ATCC type strain 700285) [42] by gavage of a single dose of 500 µl culture (generally 10^8^ organisms) as previously described [6]. Infection was confirmed 7 days post-infection (PI) by quantitative real time PCR of feces (see below). Similar numbers of helicobacter organisms were present in feces on day 7 post-infection for *Il10* ^−/−^ mice infected with 10^4^, 10^6^, or 10^8^ organisms and all infected mice developed severe colitis (data not shown). Thus the severity of colonic inflammation was insensitive to inoculum size over the range of 10^4^–10^8^ helicobacter organisms due to their rapid *in vivo* multiplication. Mice were euthanized by CO_2_ asphyxiation in accordance with the American Veterinary Medical Association Recommendations on Euthanasia if they developed 15% body weight loss, rectal prolapse, or at 25–36 wks after infection. All mice in this study were evaluated pathologically for both colitis and neoplasia.

### Tissue collection

After euthanasia, the digestive tract from stomach to anus was removed and divided into segments representing the stomach, cecum, and proximal, mid-, distal, and terminal colon/rectum. Portions of each gastrointestinal segment were rinsed briefly with PBS to remove non-adherent organisms. Tissues for molecular analysis were immediately frozen and stored at -20°C for subsequent quantitation of mRNAs or associated *Helicobacter* organisms by quantitative real-time PCR. The remaining tissues were fixed in Carnoy's solution for 2–4 hrs, then processed and embedded into paraffin.

### Histological Scoring

The severity of colonic inflammation and incidence of colon neoplasia seen in hematoxylin and eosin-stained sections was scored by a board-certified pathologist blinded to experimental group. Histologic scores were calculated as described ([43]; modified from [44]), using a scale that takes into account mucosal changes in 5 different bowel segments, including hyperplasia and ulceration, degree of inflammation, and % of each bowel segment affected by these changes. Using this scale, the maximum score is 75 and a score >12 indicates the presence of colitis.

Neoplastic lesions were classified according to a consensus report for intestinal neoplasia in mouse models as gastrointestinal intraepithelial neoplasia or invasive carcinoma [45]. The category of gastrointestinal intraepithelial neoplasia is synonymous with atypical hyperplasia, atypia, microadenoma, carcinoma *in situ*, and dysplasia, which are non-invasive neoplastic lesions. A diagnosis of invasive carcinoma required the presence of a desmoplastic response to differentiate invasion from mucosal herniation or pseudoinvasion. Regions of neoplasia that were separated by regions of normal mucosa were scored as separate lesions.

### Real-time PCR Assays

For detection of helicobacter organisms, DNA was extracted from 40 mg frozen tissue or 20 mg feces using the DNeasy Tissue kit (Qiagen, Valencia, CA). Real-time PCR was performed to quantify the relative concentrations of fecal and mucosa-associated *H. rodentium* and *H. typhlonius* organisms per milligram of feces or tissue based on comparison with a standard, as described previously [6].

For analysis of cytokine expression, total RNA was extracted from ∼30 mg cecal tissue using the RNA extraction kit (Ambion, Austin, TX). Complementary DNA (cDNA) was synthesized using 1 *μ*g of RNA through a reverse transcription reaction (Applied Biosystems, Foster City, CA). Real-time PCR quantitative mRNA analyses were performed using SYBR green fluorescence (Stratagene, Cedar Creek, TX). The standard PCR conditions were 95°C for 10 min, 40 cycles for 1 min at 94°C, 56°C for 1 min and 72°C for 2 min, followed by the standard denaturation curve. The primer sequences used in this analysis were modified from Cardoso *et al*. [46] and were as follows: *β*-actin (fwd, 5′-AGT TGC GTT TTA CAC CCT TT-3′; rev, 5′-AAG CCA TGC CAA TGT TGT CT-3′), IL-4 (fwd, 5′-CTG ACG GCA CAG AGC TAT TGA-3′; rev, 5′-TAT GCG AAG CAC CTT GGA AGC-3′), TNF (fwd, 5′-TGT GCT CAG AGC TTT CAA CAA-3′; rev, 5′-CTT GAT GGT GGT GCA TGA GA-3′), IL-12p40 (fwd, 5′-AGC ACC AGC TTC TTC ATC AGG-3′; rev, 5′-GCG CTG GAT TCG AAC AAA G- 3′), IFN-γ (fwd,5′-GCA TCT TGG CTT TGC AGC T-3′ ; rev, 5′-CTT TTT TCG CCT TGC TGT TG-3′). SYBR Green PCR Master Mix (Stratagene, Cedar Creek, TX), specific primers, and cDNA template were used in each reaction. The relative mRNA amount of each sample was calculated based on its threshold cycle, Ct, in comparison to the Ct of housekeeping gene *β*-actin. The results for cytokine mRNAs were demonstrated as mRNA expression relative to non-infected mice of the same genotype. The purity of amplified product was determined as a single peak of dissociation curve. Negative controls without RNA were also performed.

### Culture and In vitro Stimulation of Mast Cells

To obtain BMMC, mouse femurs were flushed with RPMI 1640 media using a 22G needle and 10 ml syringe. The bone marrow cells were cultured for 4 weeks in RPMI 1640 (Invitrogen, Carlsbad, CA) containing 10% fetal bovine serum, essential and non-essential amino acids, 100 U/ml penicillin, 100 µg/ml streptomycin, 10 mM HEPES, 5.5×10^−5^ M 2-mercaptoethanol, 10 ng/ml stem cell factor, and 5 ng/ml IL-3 (R&D Systems, Minneapolis, MN) to stimulate mast cell differentiation. Non-adherent cells were harvested to flasks containing fresh media once weekly. The percentage of mast cells was determined by performing a differential on a cytocentrifuge preparation stained with a modified Wright-Giemsa stain; % of mast cells averaged 95%. 10^7^ BMMC in 100 µl PBS were injected intraperitoneally to reconstitute mast cell-deficient mice, which were used 12 wks after reconstitution.

The *in vitro* reactivity of BMMC derived from C57BL/6 wild type or *Il10* ^−/−^ mice was compared by measuring degranulation, viability, and cytokine production post-degranulation. BMMC (1×10^6^ cells/well) in Tyrode's buffer were sensitized by overnight incubation with 1 µg/ml IgE then stimulated by cross-linking with 1 µg/ml goat anti-mouse IgE (BD, San Jose, CA) or by incubation with a mixture of enteric bacterial antigens prepared as 100 mg cecal contents per ml of saline or Tyrode's buffer then filtered at 0.2 µm. Degranulation was assessed by *β*-hexoseaminidase activity using a colorimetric substrate assay as previously described [47]. Viability after degranulation was measured using a tetrazolium-based colorimetric assay (CellTiter96 Aqueous, Promega, Madison, WI). Media was harvested from some wells 4 hrs after stimulation for measurement of cytokine content using a Luminex bead-based multiplex fluorescent immunoassay (BioRad, Hercules, CA).

### Intestinal permeability

The permeability of the mucosal layer was assessed using phenolsulfonphthalein (phenol red) [48]. Under normal conditions, phenol red is not absorbed in the intestine. However under conditions of increased intestinal permeability, the dye is absorbed then excreted in the urine. The urinary dye recovery was measured after administration of 2 µmol (∼700 µg) phenol red in 0.5 ml of saline by oral gavage. Groups of 2–4 animals were placed in metabolic caging that is optimized to separate urine from feces. Urine was collected for 18 hrs, alkalinized extracts were prepared, and the phenol red content of each sample was determined spectrophotometrically at 560 nm by comparison to standards of known concentration. The urinary recovery of phenol red was expressed as percent of the dose administered.

### Statistical Analysis

Statistical comparison of groups was performed using Student's t-test. Survival rates were calculated using Kaplan-Meier test with *p*-values calculated using the log rank test. Linear regression was performed using GraphPad prism software. A value of *p*≤0.05 was considered to be significant.
